# Sex Differences in Affective Facial Reactions Are Present in Childhood

**DOI:** 10.3389/fnint.2018.00019

**Published:** 2018-05-23

**Authors:** Luigi Cattaneo, Vania Veroni, Sonia Boria, Giancarlo Tassinari, Luca Turella

**Affiliations:** ^1^Dipartimento di Neuroscienze, Biomedicina e Movimento, University of Verona, Verona, Italy; ^2^Dipartimento di Neuroscienze, University of Parma, Parma, Italy; ^3^Center for Mid/Brain Sciences, University of Trento, Trento, Italy

**Keywords:** facial electromyography, emotions, mirror neurons, sadness, empathy, imitation, infancy, development

## Abstract

Adults exposed to affective facial displays produce specific rapid facial reactions (RFRs) which are of lower intensity in males compared to females. We investigated such sex difference in a population of 60 primary school children (30 F; 30 M), aged 7–10 years. We recorded the surface electromyographic (EMG) signal from the *corrugator supercilii* and the *zygomatici* muscles, while children watched affective facial displays. Results showed the expected smiling RFR to smiling faces and the expected frowning RFR to sad faces. A systematic difference between male and female participants was observed, with boys showing less ample EMG responses than age-matched girls. We demonstrate that sex differences in the somatic component of affective motor patterns are present also in childhood.

## Introduction

In the general population, males show lesser empathic personality traits compared to females. This observation has been well-documented in children and adults to the point that some authors define the male brain by its lesser empathic capacities than the female one (Baron-Cohen, 2002; Lawrence et al., 2004; Coll et al., 2012; Michalska et al., 2013). This sexual dimorphism in humans is supposed to be the product of evolutionary pressure related to mating and parental behaviors, i.e., parental investment is much greater for the female sex then for the male sex (Decety and Svetlova, 2012). Measuring empathy is challenging. Explicit self-reports by means of validated questionnaires represent the gold-standard for empathy assessment (Lawrence et al., 2004), which may be supported by a constellation of ancillary physiological (Dimberg et al., 2002) and imaging (Michalska et al., 2013) measures. Physiological measures of empathy generally consist in recording affective motor responses to other’s emotional states. The general rationale for this is rooted in the “affective” theory of empathy (Lawrence et al., 2004) that defines empathy as the capacity to re-enact other’s affective states.

Affective motor patterns recruit specific visceral and somatic effectors. Visceral responses (e.g., an increase in heart rate or a change in sweat production) are produced for homeostasis of internal functions, in contrast, somatic affective movements have a communicative role (e.g., smiling or yelling). One of the most studied somatic components of affective movements is facial expression. It has been described that individuals exposed to affective displays of conspecifics produce stereotypical facial movements that are specific to the facial expression that is observed. These movements are a robust and replicated finding and are referred to as rapid facial reactions (RFRs; Dimberg, 1982; Thunberg and Dimberg, 2000; Larsen et al., 2003; Weyers et al., 2009; Fujimura et al., 2010; Rymarczyk et al., 2011; Likowski et al., 2012); for a review see Cattaneo and Pavesi (2014). They may follow a mimetic pattern (as in smiling in response to a smile) or a reactive pattern (as in producing a fearful facial posture in response to an angry face) to the emotional facial expression of the person being observed (Dimberg, 1982; Moody et al., 2007). Subjects are unaware of own RFRs, which are not modified by superimposed voluntary movements. Another characteristic of RFRs is their sub-second onset latency, which has been documented in the 300–700 ms range (Dimberg et al., 2000). According to some authors, RFRs initiate or modulate affective states in the observer and therefore are potentially a fundamental link in the chain of events that mediate inter-individual affective communication (Dimberg et al., 2002; Larsen et al., 2003; McIntosh et al., 2006; Niedenthal et al., 2010). Consequently RFRs have been used as a tool to investigate empathy in special populations such as people diagnosed with autistic spectrum disorder (Clarke et al., 2005; McIntosh et al., 2006; Beall et al., 2008) or in particular personality traits (Scarpazza et al., 2018). Since their early descriptions, it has been shown that RFRs are unevenly produced between the two sexes (Dimberg and Lundquist, 1990; Lang et al., 1993; Thunberg and Dimberg, 2000; Sonnby-Borgstrom et al., 2008; Hermans et al., 2009; Huang and Hu, 2009; Neufeld et al., 2016). Males produce RFRs of smaller amplitude than females, possibly due to the greater female parental investment and empathy compared with males. RFRs have been recorded in children (McIntosh et al., 2006; Beall et al., 2008; Oberman et al., 2009; Deschamps et al., 2012, 2014; Geangu et al., 2016). A very large and recent web-based study confirmed sex differences in facial reactions to affective stimuli, and suggested that the differences between sexes could not be entirely quantitative (females more than males). On the contrary, in adults, qualitative differences are found, with women being more reactive to positive emotional stimuli and vice-versa, males being more reactive on negative stimuli (McDuff et al., 2017). Up to now the issue of sex differences in RFRs has not been explicitly investigated in children, and only a partial report in pre-pubertal children is available in the literature (McManis et al., 2001). We aim to fill this gap in current knowledge with the present report.

## Materials and Methods

### Participants

We examined a group of 60 typically-developing children (30 males and 30 females; age: 7; 8–10; 4 years) randomly recruited among the pupils of a local public primary school in Parma (Italy). They had no history of pre-term birth, neurodevelopmental disorders and were developing typically. Ethnicity was at 95% Caucasian, compatibly with the general Italian population^1^. All participants had normal or corrected to normal vision. Written informed consent was obtained from each participant’s parent and full approval of the child was obtained before the session. The study had been approved by the local ethical committee (Commitee for human experimentation of the University of Parma).

### Visual Stimuli

**Figure 1:**
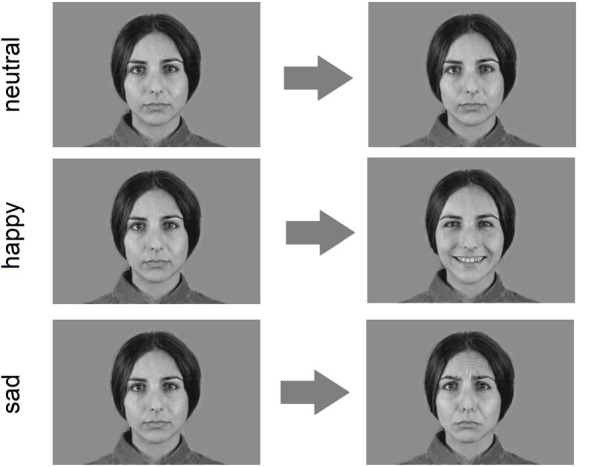
Example of experimental clips (only the first and the last frame are shown).

Visual stimuli were videos of faces passing from an initial neutral expression to full-fledged emotional expressions. We built the movies starting from grayscale photographs (14.5 cm high × 21 cm wide) selected from the Montreal Set of Facial Displays of Emotion (Beaupré and Hess, 2005) representing neutral, happy and sad expressions of Caucasian, Asian and African males and females. For each actor the neutral face was morphed by means of morphing software (*Sqirlz Morph 1*) into the full emotion in steps of 10%. In this way we obtained for each actor and emotion, 11 frames representing the spectrum of facial expression form neutral to 100% of the emotion. All frames were mounted in sequence to obtain a short video clip of 2930 ms. The clip in the neutral condition was done with a static sequence of 11 frames of the neutral face. The three stimulus types, happy, sad and neutral are shown in Figure 1. The ultimate number of stimuli were 2 (actor gender) × 3 (actor ethnicity) × 3 (emotions) × 2 (different actors within each category) = 36 clips. These were presented on a 15.4″ computer screen placed about 60 cm away from the participant.

### Procedure

Participants were greeted by the experimenters, were shown the recording laboratory and allowed time to familiarize with the environment and the equipment. They were shown the recording electrodes and were allowed to handle them. In order to avoid any bias in the participants, we avoided any reference to facial movements and muscles during the initial briefing. Instead participants were told that we were measuring skin temperature from various parts of the body and a few fake electrodes were attached to the skin of the hand and feet (Dimberg and Thunberg, 1998). Trials were initiated manually by the experimenter. The experimental session was divided in two blocks. In the first block, named “passive observation” the participants were only asked to watch attentively the clips. In the second block (“explicit rating”) they were asked to name the emotion expressed in each clip. Verbal responses were logged for further analysis. Each of the two blocks consisted in 36 stimuli, 12 clips representing happy expressions, 12 clips representing sad expressions and 12 clips of neutral expression. At the end of the session we asked all participants to perform maximal contractions of the two recorded facial muscles. This measure was used during data processing for between-subjects normalization.

### EMG Recordings, Pre-processing and Analysis

Facial EMG was recorded from the *zygomatici* muscles and the *corrugator supercilii* bilaterally with Ag/AgCl electrodes (recording area of 28 mm^2^). Muscle activity was continuously recorded using an electroencephalograph (Micromed-system, Italy), amplified ×1000 and digitized at a sampling rate of 512 Hz. The EMG signal was band-pass filtered (20–250 Hz) and rectified. The rectified EMG signal was then processed in the following steps: (1) all EMG values were divided by the EMG recorded during maximal contraction to improve comparability between subjects. EMG data was therefore expressed as percentage of maximal contraction rather than as absolute values. (2) Continuous EMG recordings were parsed into trials by considering the window from −800 ms to +2800 ms from stimulus onset. (3) Data were averaged in 400 ms consecutive bins, thus obtaining nine consecutive values of EMG for each trial. (4) The two pre-stimulus bins were averaged to create a baseline EMG value. (5) The seven post-stimulus bins were baseline-corrected by subtracting from them the baseline values. In the resulting data, any positive value indicated increased EMG activity compared to baseline and negative values indicated decreased EMG activity compared to baseline. (6) EMG data from each bin were averaged within subjects according to the emotion displayed. The resulting dataset included for each subject a total of six (2 muscles × 2 sides × 3 emotions) series of seven bins each.

### Statistical Analysis of EMG Data

The processed EMG data were analyzed by means of a mixed-design ANOVA with one between-subjects factor, SEX (2 levels: male or female) and four within-subjects factors, SIDE (2 levels: left or right) MUSCLE (2 levels: *corrugator supercilii* and *zygomatici*), EMOTION (3 levels: neutral, happy and sad) and BIN (7 levels, corresponding to each of the post-stimulus bins). *Post hoc* comparisons were made with *t-tests* with Bonferroni correction where appropriate. Greenhouse-Geisser (GG) correction was performed when appropriate. Effect size was expressed by means of the eta-squared coefficient. All statistics were performed with the STATISTICA software (StatSoft Inc.).

### Analysis of Behavioral Data

The explicit assessment of emotional expressions was classified offline into five macro-emotional categories (happiness, sadness, anger, disgust and fear). Accuracy was calculated as the ratio of correct responses on total responses. Statistical analysis on accuracy was performed by a mixed-design ANOVA with one between-subjects factor, SEX (2 levels: male or female) and one within-subjects factor, EMOTION (2 levels: happy and sad) *Post hoc* comparisons were made with *t*-tests with Bonferroni correction where appropriate.

## Results

### EMG

As a preliminary analysis, we looked for inhomogeneity in the pool of stimuli. In particular, we inspected qualitatively the EMG responses to single stimuli to identify if any were systematically more or less evocative then others in producing RFRs. We assessed quantitatively by means of a 4-way ANOVA (STIMULUS SEX*STIMULUS ETHNICITY*EMOTION*PARTICIPANT SEX) whether any effect of ethnicity or sex of the stimulus were evident. No main effects nor interactions involving STIMULUS SEX and STIMULUS ETHNICITY were evident (all *p*-values > 0.18).

**Figure 2:**
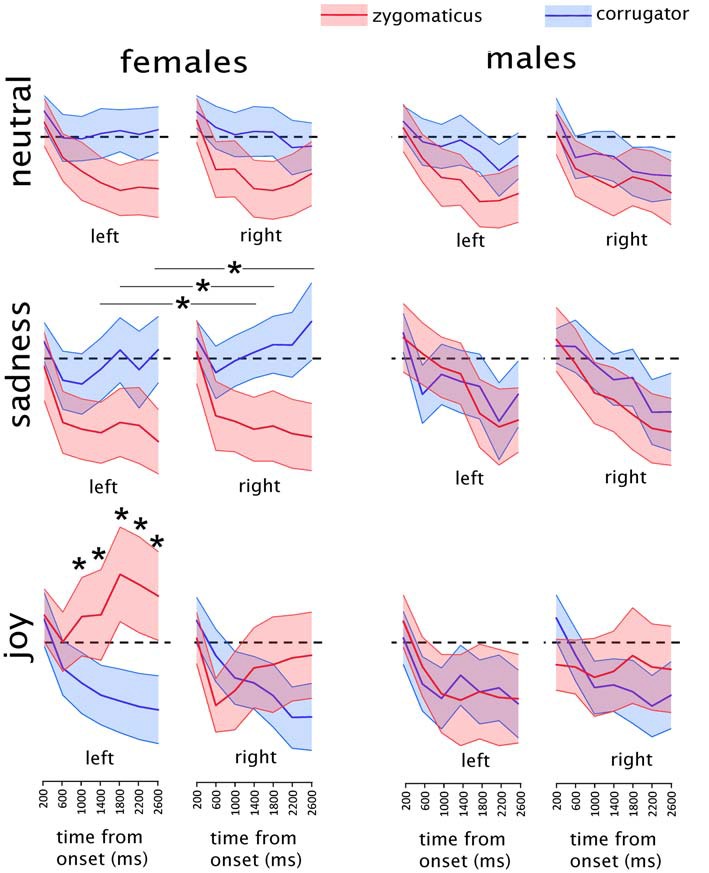
Mean results of the electromyographic (EMG) recordings in males and females in each experimental condition. Asterisks indicate the time-bins in which a significant difference was found between the two muscles. In the “sadness” condition, asterisks are represented associated with a line because they refer to the data collapsed between the two sides. Shaded areas represent the region included in the upper and lower 95% confidence intervals of the mean. The EMG activity is expressed as the percentage of maximal EMG activity, baseline corrected by subtraction of the pre-stimulus EMG (see “Materials and Methods” section for details on EMG pre-processing). Dashed black lines indicate the value of *y* = 0, corresponding to the baseline pre-stimulus EMG.

EMG results of the main analysis are illustrated in Figure 2. Given the ad-hoc interest for specific muscular responses to specific emotions, we considered as results of interest only the interactions involving SEX, MUSCLE and EMOTION. Other effects, for example a main effect of MUSCLE, are not informative for the present work and will not be discussed. The omnibus ANOVA showed a significant complex interaction of SEX*SIDE*MUSCLE*EMOTION*BIN (*F*_(12,696)_ = 3.6893, *p* = 0.00002; GG-adjusted *p* = 0.0004; eta-squared = 0.06). To investigate this 5-way interaction we split the data among the two populations of females and males into two SIDE*MUSCLE* EMOTION *BIN ANOVAs.

#### Male Group

The sub-ANOVA on male participants did not show any significant interaction of interest, i.e., involving MUSCLE and EMOTION (all *p* values > 0.12). The remaining exploratory analysis was therefore limited to the female participants.

#### Female Group

In the female group a significant interaction of MUSCLE*EMOTION*BIN (*F*_(12,348)_ = 10.846, *p* < 0.000001; GG-adjusted *p* < 0.000001; eta-squared = 0.27) was found, without any effect of SIDE. Data from the female group were further split into three separate ANOVAs, one for each of the three stimulus types (happy, sad and neutral).

##### Female Group, Happiness Clips

The analysis on happiness clips showed a significant SIDE*MUSCLE*BIN interaction: (*F*_(6,174)_ = 12.147, *p* < 0.000001; GG-adjusted *p* < 0.000001; eta-squared = 0.30). This interaction was further explored by means of two MUSCLE*BIN interactions, one for each side.

##### Female Group, Happiness Clips, Right Side

The ANOVA on the right side showed a significant MUSCLE*BIN interaction (*F*_(6,174)_ = 6.882, *p* = 0.000001); GG-adjusted *p* = 0.0001; eta-squared = 0.19). In the *Post hoc* analyses, significantly different values between the two muscles were found at the 400–800 ms bin (*t*_(29)_ = 2.1; *p* = 0.04), at the 2000–2400 ms bin (*t*_(29)_ = −2.2; *p* = 0.04) and at the 2400–2800 ms bin (*t*_(29)_ = −2.1; *p* = 0.04). However, none of them were below the adjusted p-threshold for seven multiple comparisons (*p* = 0.007) and therefore will not be considered further.

##### Female Group, Happiness Clips, Left Side

The ANOVA on the left side showed a significant MUSCLE*BIN interaction (*F*_(6,174)_ = 11.421, *p* < 0.000001; GG-adjusted *p* < 0.000001; eta-squared = 0.28). *Post hoc* tests were performed by paired-sample *t-tests* comparing values from the two muscles in the seven different bins. Given the seven comparisons, the *p-value* threshold was Bonferroni-adjusted to *p* = 0.007. Significantly different values between the two muscles were found at the 1200–1600 ms bin (*t*_(29)_ = −2.90; *p* = 0.006), at the 1600–2000 ms bin (*t*_(29)_ = −3.99; *p* = 0.0004), at the 2000–2400 ms bin (*t*_(29)_ = −3.75; *p* = 0.0007) and at the 2400–2800 ms bin (*t*_(29)_ = −3.67; *p* = 0.0009).

##### Female Group, Sadness Clips

The analysis on sadness clips showed a significant MUSCLE*BIN interaction (*F*_(6,174)_ = 5.4237, *p* = 0.00004; GG-adjusted *p* = 0.004; eta-squared = 0.16) without any effect on side. *Post hoc* tests were performed by paired-sample *t-tests* comparing values from the two muscles in the seven different bins. Given the seven comparisons, the *p-value* threshold was Bonferroni-adjusted to *p* = 0.007. Significantly different values between the two muscles were found at the 1200–1600 ms bin (*t*_(29)_ = 3.091; *p* = 0.004), at the 1600–2000 ms bin (*t*_(29)_ = 3.10; *p* = 0.004) and at the 2400–2800 ms bin (*t*_(29)_ = 3.85; *p* = 0.0006).

##### Female Group, Neutral Clips

The analysis on neutral clips showed a MUSCLE*BIN interaction (*F*_(6,174)_ = 2.2220, *p* = 0.043, eta-squared = 0.07), which did not survive the GG-adjustment (adjusted *p* = 0.07) and therefore will not be considered any further.

### Explicit Emotion Recognition

**Figure 3:**
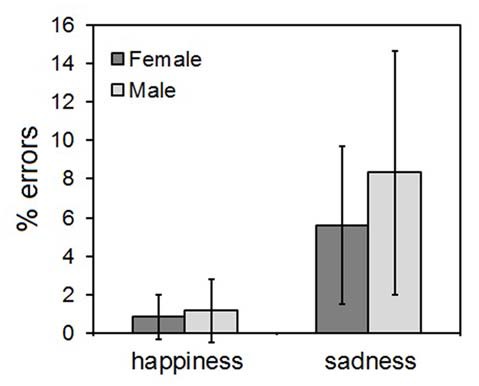
Results of the explicit emotional ratings. Error bars indicate 95% confidence interval of the mean.

The ANOVA yielded only a main effect of the CLIP factor, indicating that more errors were made in evaluating the sadness than the happiness ones (*F*_(1,58)_ = 8.44, *p* = 0.003). The types of wrong responses to the sadness clips fell into two main categories: disgust (41% of errors) and fear (37% of errors). No significant main effect of SEX (*F*_(1,58)_ = 0.59, *p* = 0.41) nor any significant SEX*EMOTION interaction (*F*_(1,58)_ = 0.28, *p* = 0.54) were found. The results are illustrated in Figure 3.

## Discussion

### Main Finding: Females Show More RFRs Than Males

The present study shows that in a population of Italian children aged 7–10 years, RFRs to affective facial displays can be readily elicited only in female subjects similarly to what has been reported in the literature for adults (Dimberg and Lundquist, 1990; Thunberg and Dimberg, 2000; Sonnby-Borgstrom et al., 2008; Hermans et al., 2009; Huang and Hu, 2009). As in adults, RFRs are evident in the EMG activity within one second, with a progressive increase of activity of the zygomatici muscles and decrease of activity of the corrugator supercilii upon showing happy faces, and vice versa an increase of activity of the corrugator supercilii and a decrease of activity of the zygomatici muscles upon showing sad faces. This pattern is more evident for the happy than the sad stimuli, that is in keeping with the only significant difference in the behavioral data, i.e., a larger number of errors with sad than happy faces.

### The Possible Communicative Role of RFRs, Beyond Mere Mimicry

What do these data tell us about behavioral differences between males and females? To answer this question one should know what psychological process are RFRs a marker of. Unfortunately, we are far from a univocal interpretation of RFRs as a biomarker of internal processes. The hypothesis that they represent a mere mimicry of observed affective displays is simplistic, therefore rendering less likely the possibility that RFRs are a marker of “empathic” capacities. The current evidence seems to point at a broader communicative role of RFRs. Facial expressions are considered to be evolutionarily shaped as a non-verbal communicative tool for conspecifics (Darwin, 1872; Ekman and Oster, 1979). In this context, RFRs are often referred to as mimicry of emotional activity. However, RFRs seem to be more complex patterns of affective motor response than mere mimicry. They represent affective responses to certain evolutionary/biologically relevant stimuli, which can be facial expressions just as well as snakes or spiders (Cacioppo et al., 1986; Dimberg, 1986). In fact, RFRs are readily elicited by affectively charged scenes in the absence of facial displays and therefore their potential role in the reception of communicative affective signals is weak. Moreover, when RFRs are produced in response to facial displays, they do not necessarily follow a mimetic pattern, but can be reactive (as in producing a fear expression in response to an angry face) (Moody et al., 2007). Taken together, these data seem to point at a communicative role of RFRs that is more tuned to sending information to conspecifics rather than receiving information from conspecifics. In the light of this assumption, the present data less likely indicate a “less empathic” brain in our population of male children but more likely a lesser attitude to communicate within-group socially relevant affective information. At least in part it possible to infer attitudes from facial EMG response. The impact of social context in mimicry has been studied using facial EMG responses by Bourgeois and Hess (2008), who showed that the level of facial mimicry varies as a function of group membership.

### Acquired or Innate Origin of Sex Differences?

Finding neuro-behavioral differences in developmental age raises the question whether we are describing a genetically-determined, innate pattern or a pattern acquired during early development because of interaction with the environment. We know from the literature that RFRs are present in infancy in children as young as 6 years (Deschamps et al., 2012) and it has been recently shown (Geangu et al., 2016) that 3-year-old children already display RFRs to faces, but not to bodily emotion expressions, unlike adults, in whom RFRs elicited both by facial and body emotions (Magnée et al., 2007). The presence of RFRs in 3-year-old children seem to point out at a hard-wired mechanism at their origin. On the contrary, we cannot speculate in any way about the innate or acquired origin of the sex difference found by us, nor we think our study is appropriately designed to test such hypothesis. Indeed, several culturally-mediated factors, alien to basic emotional processing, can modulate RFRs. For instance recently formed negative or positive attitudes toward the observed actors can invert RFR patterns (Likowski et al., 2008). RFRs may be modulated by the attribution of fairness to the actor (Hofman et al., 2012). Facial expression is strongly influenced by a social contextual effects (Vrana and Gross, 2004). In particular group membership can modulate RFRs, which tend to be more evident in response to in-group faces (Yabar et al., 2006). Finally, also task-related factors could be at the source of the observed difference between male and female participants. Performance anxiety can modulate RFRs (Dimberg and Thunberg, 2007). Also perceived task difficulty and task demands can generate RFR-like responses. In fact it has been shown that frowning-like RFRs can contaminate responses to positive affective displays if a task is resource-demanding (Lishner et al., 2008). In the light of these observations, any difference in human environment surrounding boys or girls could have affected their early personality traits. Nevertheless, the present finding is robust and it has considerable implications for further research, especially from the methodological point of view, as outlined in the following paragraph.

### Value of the Present Data for RFR Research in Human Development

Whatever the cause, present data are rather explicit in telling that sex differences in such capacities are already expressed in the age bracket of the present study and therefore they are of considerable value at least at a methodological level. RFRs are being increasingly used to test pathogenetic hypotheses on neurodevelopmental disorders (McIntosh et al., 2006; Press et al., 2010; Deschamps et al., 2014), and are therefore increasingly used in pediatric populations. The original finding of sex differences in RFRs in adults (Thunberg and Dimberg, 2000) greatly influenced the selection of experimental populations in the subsequent literature. We believe that a similar finding in children should prompt similar caution in testing developmental disorders.

## Conclusion

We found that the peculiar sexual dimorphism in RFRs that is well-documented in adults is already present at pre-pubertal age. Seven to ten year old males showed lesser EMG activity in facial muscles than their female peers. Uncertainty on the functional role of RFRs limits the interpretation of the data. Embodied cognition theories postulate their role in awareness of self and others’ emotional states. RFRs may have role in non-verbal communication, similarly to overt facial affective displays. In this respect, the present data are in line with known differences between male and female affective behavior, though they do not solve the issue on whether these differences are acquired or developmental. Importantly, RFRs are generally used in research as a physiological marker of affective processes and the present finding raises relevant methodological issues in terms of population selection.

## Author Contributions

LC, VV and SB designed the study and collected data. LC and LT analyzed the data. All authors wrote the manuscript.

## Conflict of Interest Statement

The authors declare that the research was conducted in the absence of any commercial or financial relationships that could be construed as a potential conflict of interest.

## Footnotes

^1^https://www.istat.it/it/archivio/204296
